# Comparison of PCR/Electron spray Ionization-Time-of-Flight-Mass Spectrometry versus Traditional Clinical Microbiology for active surveillance of organisms contaminating high-use surfaces in a burn intensive care unit, an orthopedic ward and healthcare workers

**DOI:** 10.1186/1471-2334-12-252

**Published:** 2012-10-10

**Authors:** Heather C Yun, Rachael E Kreft, Mayra A Castillo, Garth D Ehrlich, Charles H Guymon, Helen K Crouch, Kevin K Chung, Joseph C Wenke, Joseph R Hsu, Tracy L Spirk, J William Costerton, Katrin Mende, Clinton K Murray

**Affiliations:** 1 San Antonio Military Medical Center, San Antonio, TX, USA; 2 Uniformed Services University, Bethesda, MD, USA; 3 Center for Genomic Sciences, Allegheny Singer Research Institute, Pittsburgh, PA, USA; 4 Departments of Microbiology and Immunology, and Otolaryngology Head and Neck Surgery, Drexel University College of Medicine, Pittsburgh, PA, USA; 5 US Army Institute of Surgical Research, San Antonio, TX, USA; 6 Infectious Disease Clinical Research Program, Uniformed Services University, Bethesda, MD, USA

**Keywords:** PCR/ESI-TOF-MS, Ibis, Microbiology, Contamination, Environment

## Abstract

**Background:**

Understanding nosocomial pathogen transmission is restricted by culture limitations. Novel platforms, such as PCR-based electron spray ionization-time-of-flight-mass spectrometry (ESI-TOF-MS), may be useful as investigational tools.

**Methods:**

Traditional clinical microbiology (TCM) and PCR/ESI-TOF-MS were used to recover and detect microorganisms from the hands and personal protective equipment of 10 burn intensive care unit (ICU) healthcare workers providing clinical care at a tertiary care military referral hospital. High-use environmental surfaces were assessed in 9 burn ICU and 10 orthopedic patient rooms. Clinical cultures during the study period were reviewed for pathogen comparison with investigational molecular diagnostic methods.

**Results:**

From 158 samples, 142 organisms were identified by TCM and 718 by PCR/ESI-TOF-MS. The molecular diagnostic method detected more organisms (4.5 ± 2.1 vs. 0.9 ± 0.8, p < 0.01) from 99% vs. 67% of samples (p < 0.01). TCM detected *S. aureus* in 13 samples vs. 21 by PCR/ESI-TOF-MS. Gram-negative organisms were less commonly identified than gram-positive by both methods; especially by TCM. Among all detected bacterial species, similar percentages were typical nosocomial pathogens (18-19%) for TCM vs. PCR/ESI-TOF-MS. PCR/ESI-TOF-MS also detected *mec*A in 112 samples, *van*A in 13, and KPC-3 in 2. *Mec*A was associated (p < 0.01) with codetection of coagulase negative staphylococci but not *S. aureus*. No *van*A was codetected with enterococci; one KPC-3 was detected without *Klebsiella* spp.

**Conclusions:**

In this pilot study, PCR/ESI-TOF-MS detected more organisms, especially gram-negatives, compared to TCM, but the current assay format is limited by the number of antibiotic resistance determinants it covers. Further large-scale assessments of PCR/ESI-TOF-MS for hospital surveillance are warranted.

## Background

Healthcare-associated infections (HAI) account for substantial morbidity and mortality worldwide [1]. These occur both in epidemics, with a common pathogen, and in endemic settings, where no clusters or common pathogens are identified. Numerous reservoirs for epidemiologically significant organisms have been demonstrated in healthcare settings. These include high-use environmental surfaces, such as door handles and handrails; patient care items such as bedside tables, bedrails, and intravenous fluid (IV) pumps; healthcare provider protective clothing such as lead aprons; and plumbing structures including drains and faucet heads, and computer equipment, among many others [2-7]. Contamination of personal protective equipment (PPE) during patient care is a mechanism for transient colonization in healthcare workers (HCW) after doffing PPE [8,9]. However, in any healthcare environment, identification of a reservoir for endemic transmission of pathogens is the exception rather than the rule. Identifying reservoirs is limited by the sensitivity of traditional clinical microbiology (TCM), especially since many pathogens establish biofilms, which are recalcitrant to TCM, on environmental surfaces [10,11]. More accurate identification and speciation of environmental pathogens should assist infection prevention efforts and mitigate excess patient morbidity and mortality.

Molecular techniques are increasingly used for microbial detection; however, these methods often focus on a single pathogen, such as methicillin-resistant *Staphylococcus aureus* (MRSA), or are used only after initial growth of bacteria in culture [12,13]. Ideal molecular methods would include the ability to screen samples for numerous species rapidly and simultaneously. The Ibis T5000 (PCR electron spray ionization-time-of-flight-mass spectrometry; PCR/ESI-TOF-MS) technology is based on the determination of the ratios of the four nucleotide bases (A, T, G and C) in multiple (n = 16) PCR amplicons that target conserved bacterial genes (including the 16S rDNA gene). Using a triangulation algorithm based on multiple independent amplicon mass determinations, it can identify and speciate all eubacterial species present in a complex sample that are present at greater than 3% of the microbial burden [14]. The technology has been recently reviewed in detail [15-17]. It has been used in outbreak investigations of *Streptococcus pyogenes* and *Acinetobacter* spp., to characterize and genotype a diverse collection of *S. aureus* isolates, and to characterize orthopedic infections [18-23]. However, no previous study using this technology has evaluated recovery of endemic pathogens in a healthcare environment. This pilot study uses TCM and PCR/ESI-TOF-MS to compare contamination of HCW hands and PPE used in the care of patients on the burn intensive care unit (ICU), and contamination of high-use surfaces in the burn ICU and the orthopedic ward. Additionally, we explored whether results obtained from either TCM or PCR/ESI-TOF-MS reflected contemporaneous clinical cultures obtained from hospitalized patients on the study units.

## Methods

### Isolates tested

Sample acquisition was planned from 20 occupied single-bed patient care rooms, ten from the burn ICU (burn unit rooms were designed with anterooms and universal gowns and gloves are used) and ten from the orthopedic ward. Nine rooms in the burn ICU had sample acquisition completed due to patient census. In the burn ICU, one HCW for each selected patient room was also enrolled for screening. Two HCW completed patient care in the same room in one instance due to patient census. Two swabs (one for TCM and one for PCR/ESI-TOF-MS; Fisherfinest Transport Swabs with Liquid Stuarts) were obtained using a standard rolling technique from: the door handle exiting the room, sink faucet, bedrail, IV pump, in-room computer keyboard, and in-room computer mouse where available. In rooms where any of these items was unavailable, these data were omitted. Bandage shears from 10 orthopedic surgeons were also swabbed.

### HCW screening

Two swabs (Fisherfinest Transport Swabs with Liquid Stuarts) were obtained (using the standard rolling technique) from subjects’ hands. HCW donned PPE (gowns and gloves) and managed their patients in single patient room. Upon return, the surfaces of gloves, the waistline of the gown, and the hands after glove removal and before hand hygiene were swabbed. One swab was tested using TCM techniques and the other by PCR/ESI-TOF-MS.

### Clinical culture data

A summary of de-identified clinical culture and *Clostridium difficile* toxin assay results (included due to its significance as a HAI bacterial pathogen, inability to isolate by routine clinical culture, and in order to correlate against any PCR/ESI-MS-TOF *C. difficile* results obtained) obtained during routine patient care from the burn ICU and orthopedics ward during the study period was retrospectively collated via the patient’s electronic medical records. Clinical cultures (and *C. difficile* toxin assay results) were included if performed from t-14 through t + 14 days with respect to the dates of room sampling for that unit, which took place from May-July 2010. No concurrent chart review was performed for hospital length of stay, definitions of infections, or any other clinical criteria since the hospital microbial ecology was the outcome of interest, and no potentially duplicate isolates from the same patient were excluded. Organisms were considered potentially clinically relevant if isolated on at least five occasions from separate clinical cultures during the study period and they were not common skin contaminants. For the purposes of statistical comparisons, coagulase negative staphylococci (CNS) were excluded, and aerobic gram-negative rods other than *Escherichia coli*, *Acinetobacter* spp.*, Klebsiella* spp.*, Pseudomonas* spp., or *Enterobacter* spp. were coalesced into one category.

### PCR/ESI-TOF-MS

Methods for genotypic characterization of bacterial and fungal isolates, and genetic resistance elements (*mec*A, *van*A, and KPC-3) using the commercially available Ibis T5000 (Ibis Biosciences) have been described elsewhere [21,24]. Swabs were frozen at −80°C and shipped on dry ice for batched PCR/ESI-TOF-MS testing. Following thawing of the swabs they were placed into sterile microcentrifuge tubes containing 270 μl of ATL Lysis buffer (Qiagen, Germantown, MD, cat# 19076) and 30 μl proteinase K (Qiagen, cat# 19131). Samples were incubated at 56°C for one hour. One hundred μl of a mixture containing 50 μl each of 0.1 mm and 0.7 mm Zirconia beads (Biospec cat# 11079101z, 11079107zx respectively) were added to the samples which were then homogenized for 10 min at 25 Hz using a Qiagen Tissuelyser. Nucleic acid from the lysed sample was then extracted using the Qiagen DNeasy kit (Qiagen cat# 69506). 10 μl of each sample was loaded per well onto the BAC detection PCR plate (Abbott Molecular, cat# PN 05 N13-01). The BAC detection plate is a 96 well plate which contains 16 primers that survey all bacterial organisms by using multiple omnipresent loci (e.g. 16S rDNA sequences) and multiple pluripresent loci (e.g. the *tuf*B gene). This has been validated against 613 organisms, meaning it correctly identified them when presented with unknowns. The system also detects the presence of several key antibiotic resistance markers: *van*A and *van*B (vancomycin resistance) in *Enterococcus* spp., KPC-3 (carbapenem resistance) in gram-negative bacteria, and *mec*A (methicillin resistance) in *Staphylococcus* spp. An internal calibrant of synthetic nucleic acid template is also included in each assay, controlling for false negatives (e.g. from PCR inhibitors) and enabling a semi-quantitative analysis of the amount of template DNA present. PCR amplification was carried out as per Ecker et al [25]. The PCR products were then desalted in a 96-well plate format and sequentially electrosprayed into a time-of-flight mass spectrometer. The spectral signals were processed to determine the masses of each of the PCR products present with sufficient accuracy that the base composition of each amplicon could be unambiguously deduced. Using combined base compositions from multiple PCRs, the identities of the pathogens and a semi-quantitative determination of their relative concentrations in the starting sample were established by using a proprietary algorithm to interface with the Ibis database of known organisms.

Semi-quantitative data was obtained from all PCR/ESI-TOF-MS analyses as each well of each assay is seeded with a DNA template that contains the appropriate primer binding sites for the primers in that well. These primer binding sites flank a synthetic DNA sequence of known composition. By comparing the amount of each species’ amplimer produced in a well to the amount of the amplimer resulting from the synthetic template the number of genomes/well of each bacterial species can be approximated. However, given the exploratory nature of the study, semi-quantitative data were not analyzed here.

### TCM

Clinical microbiology swabs were transferred to brain heart infusion (BHI) broth medium and this was incubated 48 h at 35–37°C. If the BHI demonstrated turbidity, the inoculated broth was subcultured onto sheep’s blood agar plates (BBL, Cockeysville, MD, USA) and MacConkey agar plates (BBL, Cockeysville, MD, USA). All colony forming units were worked up with no minimum threshold for evaluation. Organisms and antimicrobial resistance testing were performed using standard clinical microbiology techniques including semi-automated mechanisms for gram-negative isolates (Siemens WalkAway 40 System; Siemens Healthcare Diagnostics, Deerfield, IN, USA).

### Human subject protection

The protocol was reviewed and approved by the Brooke Army Medical Center Institutional Review Board and human subjects provided informed consent.

### Statistical analysis

Descriptive statistics were used to summarize findings. Analysis was performed using existing software (SPSS, version 19.0; IBM SPSS). Categorical variables were compared by chi-squared test, and *t*-test for normal continuous variables. Paired tests were applied when comparing two methods of testing from the same sample; McNemar’s test was used for nonparametric paired testing. Means and standard deviations are expressed throughout as mean ± SD. All p-values are two-tailed and statistical significance represented by p < 0.05.

## Results

Samples were taken from 158 sites; 40 from HCW (10 pre-patient care hands, 10 gloves, 10 gowns, 10 post-patient care hands), 19 from door handles, sink faucets, IV pumps and bedrails, 17 from keyboards, 15 from computer mice, and 10 from orthopedic shears. From these sites, 142 organisms were recovered by TCM and 718 by PCR/ESI-TOF-MS. At all sites, compared to TCM, PCR/ESI-TOF-MS recovered a larger number of organisms (4.5 ± 2.1 vs. 0.9 ±0.8, p <0.01) from a greater proportion of samples (99% vs. 67%, p <0.01; Table 1). HCW hands revealed more organisms by PCR/ESI-TOF-MS than TCM before care (3.9 ± 2.0 vs. 0.4 ± 0.5, p < 0.01) and after care (3.8 ±1.6 vs. 0.6 ± 0.5, p < 0.01). PCR/ESI-TOF-MS also recovered a greater number of organisms than TCM among used gowns (2.8 ± 1.1 vs. 0.6 ± 0.7, p < 0.01), but not gloves (3.1 ± 2.3 vs. 1.3 ± 1.6, p = 0.10).

**Table 1:** PCR/Electron spray ionization-time-of-flight-mass spectrometry (PCR/ESI-TOF-MS) versus traditional clinical microbiology (TCM) for detection of organisms contaminating high-use surfaces, healthcare worker hands, and personal protective equipment in a burn intensive care unit (ICU) and an orthopedic ward

	**Burn ICU # sites with at least one organism recovered (# organisms recovered)**			**Orthopedic ward # sites with at least one organism recovered (# organisms recovered)**		
	**Screened**	**PCR/ESI-TOF-MS**	**TCM**	**Screened**	**PCR/ESI-TOF-MS**	**TCM**
Bedrails	9	9 (44)	7 (11)	10	10 (53)	8 (11)
Door handles	9	9 (34)	6 (7)	10	10 (48)	3 (3)
Sink faucets	9	9 (41)	7 (8)	10	10 (56)	9 (11)
IV pumps	9	8 (34)	6 (7)	10	10 (53)	5 (6)
Keyboards	9	9 (48)	9 (16)	8	8 (50)	8 (11)
Mouse	9	9 (38)	6 (8)	6	6 (35)	4 (6)
Shears				10	10 (48)	7 (8)
Hands pre-care	10	10 (39)	4 (4)			
Gloves	10	10 (31)	6 (13)			
Gowns	10	9 (28)	5 (6)			
Hands post-care	10	10 (38)	6 (6)			
Total	94	92 (375)	62 (86)	64	64 (343)	44 (56)

Total number of isolates recovered.

**Figure 1:**
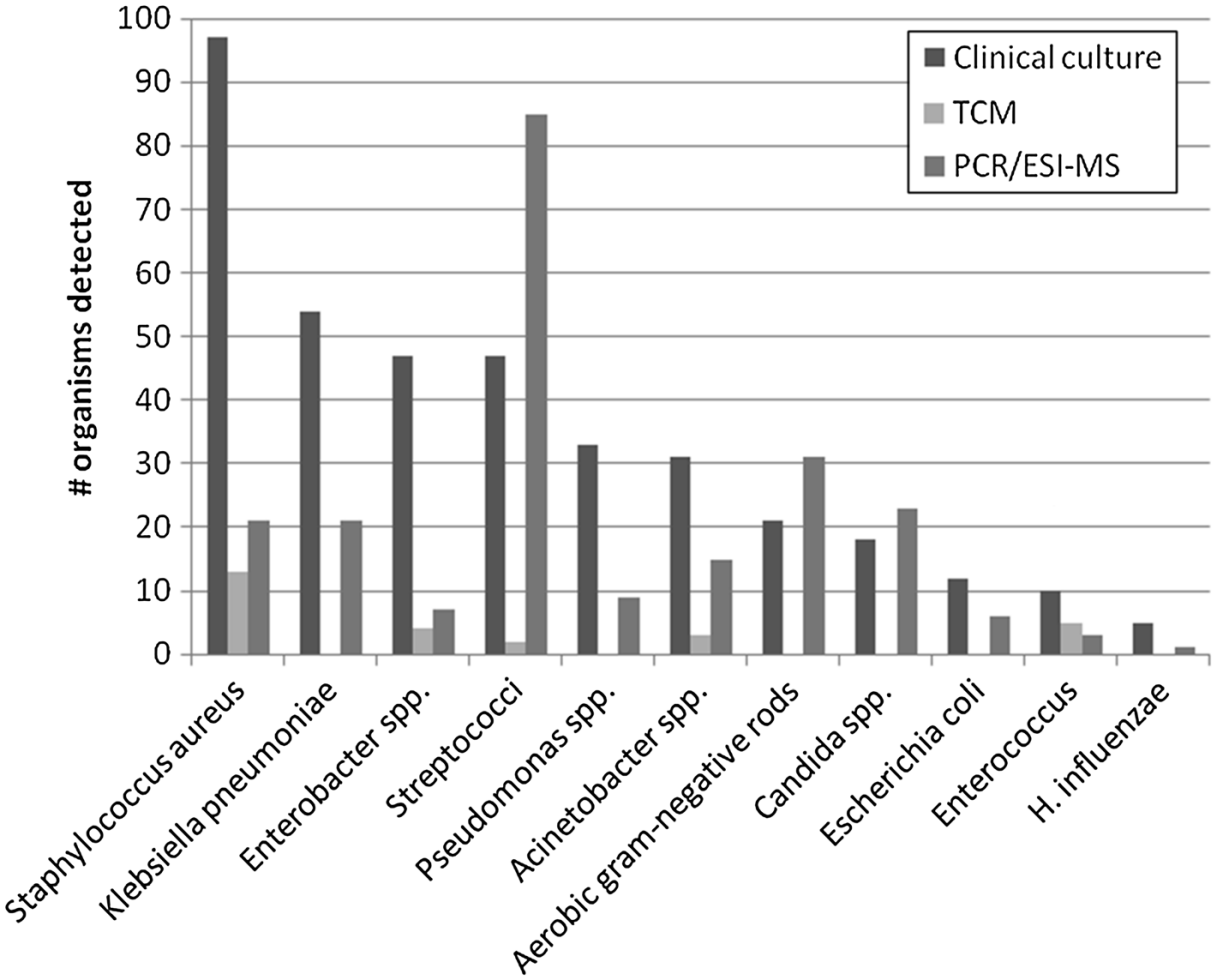
**Number of pathogens of potential clinical relevance detected by clinical culture versus numbers detected on healthcare worker hands, personal protective equipment and environmental surfaces by traditional clinical microbiology (TCM) and PCR/Electron spray ionization-time-of-flight-mass spectrometry (PCR/ESI-TOF-MS) in the burn unit and orthopedic ward.** “Aerobic gram-negative rods:” Clinical culture: 1 *Achromobacter xylosoxidans*, 1 *Aeromonas sobria*, 4 *Citrobacter koseri*, 4 *Morganella morganii*, 5 *Providencia rettgeri*, 2 *Serratia marcescens*, 8 *Stenotrophomonas maltophilia*. PCR/ESI-TOF-MS: 1 *Actinobacillus* sp., 1 *Azoarcus* sp., 4 *Bordetella avium*, 1 *Bordetella bronchiseptica*, 1 *Bordetella parapertussis*, 2 *Bordetella petri*, 2 *Burkholderia cenocepacia*, 4 *Burkholderia thailandensis*, 1 *Campylobacter* sp., 1 *Caulobacter* sp., 1 *Leptothrix cholodnii*, 1 *Nitrosomonas europaea*, 2 *Novosphingobium aromaticivorans*, 1 *Raoultella ornithinolytica*, 3 *S. marcescens*, 1. *S. flexneri*, 1 *Sphingomonas* sp., 1 *Vibrio rumoiensis*, 1 *Vibrio vulnificus*, 1 *Xanthomonas oryza.*

Organisms recovered from 393 clinical cultures included S*. aureus, Klebsiella pneumoniae, Enterobacter* spp., *Streptococcus* spp. (68% viridans group), *Pseudomonas aeruginosa*, and *Acinetobacter baumannii-calcoaceticus* complex; the proportions of these organisms detected by PCR/ESI-TOF-MS and TCM are depicted in Figure 1. The most common clinical culture sources included respiratory (29%), wound (22%), body fluid (19%), and blood (16%). There were no positive toxin assay results for *C. difficile*. Twelve isolates of CNS were recovered, 8 from blood cultures. By the study definition of potentially clinically relevant organisms, and combining less commonly recovered aerobic gram-negative rods (e.g. *Serratia, Morganella, Stenotrophomonas* spp.), 84% of clinical cultures were potentially clinically relevant. There was no difference in the proportion of potentially clinically relevant organisms detected by TCM vs. PCR/ESI-TOF-MS (18 vs. 19%, p = 0.77). Including streptococci, which were the third most commonly recovered organisms among clinical cultures, 19% of TCM organisms recovered were of potential clinical significance vs. 31% for PCR/ESI-TOF-MS (p < 0.01). Comparison of samples positive for a potentially clinically relevant organism revealed consistently higher proportions detected by PCR/ESI-TOF-MS (Table 2). This was statistically significant by McNemar’s test with or without inclusion of streptococci, and remained significant even when comparing only samples positive for the most commonly cultured bacteria (*S. aureus*, *K. pneumoniae*, *Enterobacter* spp., *Pseudomonas* spp., *Acinetobacter* spp., and *E. coli*).

**Table 2:** PCR/Electron spray ionization-time-of-flight-mass spectrometry (PCR/ESI-TOF-MS) versus traditional clinical microbiology (TCM) for detection of pathogens of potential clinical relevance on healthcare worker hands/personal protective equipment and high-use surfaces (n = 158)

		**PCR/ESI-TOF-MS # sites with at least one organism recovered (%)**	**TCM # sites with at least one organism recovered (%)**	**p value**
Any potentially clinically relevant organism*	Including streptococci	123 (77.8)	20 (12.7)	<0.01
	Not including streptococci	94 (59.4)	19 (12.8)	<0.01
Six most common bacteria recovered from clinical cultures**		58 (36.7%)	16 (10.1)	<0.01

*S. aureus, *K. pneumoniae*, *Enterobacter* spp., *Pseudomonas* spp., *Acinetobacter* spp., Aerobic gram-negative rods (see Figure 1), *Candida* spp., *E. coli*, *Enterococcus* spp., and *Haemophilus influenzae.*

**S. aureus, *K. pneumoniae*, *Enterobacter* spp., *Pseudomonas* spp., *Acinetobacter* spp., and *E. coli.*

Distribution of potentially clinically relevant organisms, plus CNS, recovered from HCW hands/PPE, and the hospital environment, are presented in Tables 3 and 4 respectively. Most organisms recovered by either mechanism were gram-positive. Eight-six total CNS isolates were recovered by TCM and 214 by PCR/ESI-TOF-MS; 13 *S. aureus* by TCM and 21 by PCR/ESI-TOF-MS. Gram-negative organisms were less commonly identified, especially by TCM. There were 3 *Acinetobacter* spp. recovered by TCM and 15 by PCR/ESI-TOF-MS; 4 *Enterobacter* spp. by TCM and 7 by PCR/ESI-TOF-MS; no *E. coli* by TCM and 6 by PCR/ESI-TOF-MS, no *Klebsiella* spp. by TCM and 21 by PCR/ESI-TOF-MS, no *Pseudomonas* spp. by TCM and 9 by PCR/ESI-TOF-MS. These five gram-negative rod species (GNR) contributed 177 of 389 (45%) clinical cultures during the study period, however only 7 (5%) of the environmental samples were positive for these organisms by TCM, and 58 (8%) by PCR/ESI-TOF-MS. TCM contributed to only 11% of total detections of these GNR, vs. 38% of all *S. aureus* detections, a difference that was statistically significant (p <0.01).

**Table 3:** PCR/Electron spray ionization-time-of-flight-mass spectrometry (PCR/ESI-TOF-MS) versus traditional clinical microbiology (TCM) for detection of most frequently recovered potentially clinically relevant pathogens, plus coagulase negative staphylococci (CNS), contaminating healthcare workers and personal protective equipment

	**PCR/ESI-TOF-MS (n = 77)**				**TCM (n = 23)**			
**Organism**	**Hands pre-care**	**Gloves**	**Gowns**	**Hands post-care**	**Hands pre-care**	**Gloves**	**Gowns**	**Hands post -care**
CNS	12	6	8	12	4	4	3	3
*Staphylococcus aureus*		3	3	1		3	1	1
Streptococci*	9	4	3	1		1		
*Acinetobacter* spp.**		3	1			1		
*Enterobacter* spp.***		1				2		
*Escherichia coli*		1	1					
*Klebsiella pneumoniae*	1	3	1	1				
*Pseudomonas* spp.****	1			1				
*Candida* spp.*****		2						

*TCM: 1 Group D *Streptococcus*; PCR/ESI-TOF-MS: 7 *S. agalactiae,* 10 viridans group streptococci.

**TCM: 1 *A. baumannii*; PCR/EIS-MS: 2 *A. baumannii,* 2 *A. calcoaceticus.*

***TCM: 2 *Enterobacter aerogenes*; PCR/ESI-TOF-MS: 1 *E. aerogenes.*

****PCR/ESI-TOF-MS: 1 *Pseudomonas fluorescens*, 1 *Pseudomonas mendocina.*

*****PCR/ESI-TOF-MS: 1 *C. albicans*, 1 *Candida glabrata.*

**Table 4:** PCR/Electron spray ionization-time-of-flight-mass spectrometry (PCR/ESI-TOF-MS) versus traditional clinical microbiology (TCM) for detection of most frequently recovered potentially clinically relevant organisms, plus coagulase negative staphylococci (CNS), contaminating high-use surfaces

**Bacteria**	**PCR/ESI-TOF-MS (# isolates) n = 305**							**TCM (# isolates) n = 89**						
	**Bedrail**	**Door handle**	**Faucet**	**IV pump**	**Keyboard**	**Mouse**	**Shears**	**Bedrail**	**Door handle**	**Faucet**	**IV pump**	**Keyboard**	**Mouse**	**Shears**
CNS	24	31	28	23	30	22	19	11	6	14	9	16	9	7
Enterococci*		2					1	2	1		1			
*S. aureus*	4		1	4	2	3		3		1	1	1	2	
Streptococci**	7	8	12	12	17	7	5							1
*Acinetobacter* spp.***	2	1	2	6					1		1			
*Enterobacter* spp.****	3	1	1	1				1	1					
*E. coli*	2	1		1										
*K. pneumoniae*	7		1	3	1	3								
*Pseudomonas* spp.*****	4		2		1									
*Candida* spp.******	7	3	5	3	1	2								

*TCM: “*Enterococcus* spp.”; PCR/ESI-TOF-MS: 2 *Enterococcus faecalis*, 1 *Enterococcus faecium.*

**TCM: 1 viridans group streptococci; PCR/ESI-TOF-MS: 1 Group D *Streptococcus*, 24 *S. agalactiae*, 8 *Streptococcus pneumoniae*, 1 *S. pyogenes*, 1 “*Streptococcus* spp.”, 33 viridans group streptococci.

***TCM: 2 *A. baumannii*; PCR/ESI-TOF-MS: 10 *A. baumannii*, 1 “*Acinetobacter* spp.”

****TCM: 2 *Enterobacter cloacae*; PCR/ESI-TOF-MS: 4 *E. cloacae*; 2 *Enterobacter sakazakii.*

*****PCR/ESI-TOF-MS: 1 *P. aeruginosa*, 2 *Pseudomonas entomophila/putida*, 3 *P. fluorescens*, 1 “*Pseudomonas* spp.”

******PCR/ESI-TOF-MS: 10 *C. albicans*, 4 *C. glabrata*, 1 *Candida kefyr*, 1 *Candida kruseii,* 1 *Candida parapsilosis*, 1 *Candida rugosa*, 3 *Candida tropicalis.*

In addition to detection of bacteria, PCR/ESI-TOF-MS detected the *mec*A gene in 112 samples. The majority of these codetected CNS with no *S. aureus* present (93; 83%). The remainder were comprised of *S. aureus* alone (9); *S. aureus* and CNS together (8), or no staphylococci (2). *Mec*A detection was statistically associated with CNS codetection (p <0.01) but not with either *S. aureus* detection or MRSA growth on culture (p = 0.22) from the same samples. Of 13 *S. aureus* isolates recovered by TCM, 9 were MRSA. Seven of 9 MRSA cultured also had *mec*A and *S. aureus* detected by PCR/ESI-TOF-MS from the same sample (one detected *mec*A with no *S. aureus* and the other detected neither). Of the two samples for which *mec*A but no staphylococci were recovered, one grew *Acinetobacter* spp. and enterococci by TCM. PCR/ESI-TOF-MS detected *A. baumannii*, *Candida albicans*, and *Polynucleobacter* spp. The other sample was TCM negative with both *P. acnes* and *Streptococcus thermophilus* detected by PCR/ESI-TOF-MS.

There were 13 samples in which *van*A was detected. None of these had enterococci codetected by PCR/ESI-TOF-MS, and no cultures grew vancomycin-resistant enterococci (VRE); one grew susceptible enterococci. There was a significant association with lactobacilli codetection; 6 of 20 lactobacillus detections had *van*A codetected (p < 0.01). However, 7 samples were positive for *van*A with neither lactobacilli nor enterococci codetections. There were no clear trends among the other organisms codetected with *van*A, but 12 of 13 *van*A samples also had a *mec*A codetected.

KPC-3 was detected in 2 samples, in one of which *K. pneumoniae* was codetected (along with *Clostridium perfringens, Saccharomyces cerevisiae, S. thermophilus*, CNS, and *mec*A). The other KPC-3 positive sample codetected CNS, *Streptococcus agalactiae, Propionibacterium acnes, Lactobacillus salivarius, Nocardia asteroides, Bordetella bronchiseptica*, and *van*A.

There were a number of rare or unexpected microorganisms detected in the hospital environment by PCR/ESI-TOF-MS, including *Shigella*, *Vibrio* and *Bartonella* spp. Selected unusual or less commonly detected organisms are presented in Table 5.

**Table 5:** Selected rare organisms detected by PCR/Electron spray ionization-time-of-flight-mass spectrometry (ESI-TOF-MS) in the hospital environment

**Microbiology**	**# PCR/ESI-TOF-MS detections**		
	**HCW/PPE**	**Burn ICU rooms**	**Orthopedic rooms/Shears**
Gram-positive			
Cocci			
* Leuconostoc* spp.	2	0	5
* S. pyogenes*			1
* S. pneumoniae*		1	7
Bacilli			
* Clostridium* spp.	1	1	2
* Clostridium tetani*		1	1
* Listeria monocytogenes*	2		
* Nocardia* spp.	3	1	6
Gram-negative			
Cocci			
* Neisseria* spp.		1	2
Aerobic Bacilli			
* Bordetella* spp.	3	1	4
* Burkholderia* spp.	1	2	3
* Polynucleobacter* spp.	7	11	7
* Shigella flexneri*			1
* Vibrio* spp.		1	1
Anaerobic Bacilli			
* Bacteroides* spp.	1	1	
Other bacteria			
* Bartonella* spp.	1		
* Borrelia turicatae*			1
* Mycobacterium abscessus*			1
* Mycoplasma hominis*	1		
Fungi			
* Alternaria*	2	1	4
* Saccharomyces cerevisiae*	1	3	17
Other fungi*		1	3

*HCW* healthcare worker, *PPE* personal protective equipment, *ICU* intensive care unit.

* One of each: *Botryosphaeria rhodina*, *Macroventuria* spp., *Cochliobolus* spp., *Arthrographis cuboidea.*

Few clinically relevant pathogens were detected by TCM and not by PCR/ESI-TOF-MS. Altogether, PCR/ESI-TOF-MS failed to detect 35 of the 142 isolates from TCM, most of which were identified as *Micrococcus* spp. and CNS. There were five *Enterococcus* spp. isolated by TCM which went undetected by PCR/ESI-TOF-MS; additional clinically relevant organisms included *S. aureus* (2), *Enterobacter* spp. (2), and *Acinetobacter* spp. (1).

## Discussion

Endemic transmission of nosocomial pathogens, especially gram-negative organisms, in a hospital environment is often poorly defined and difficult to control. While some studies have demonstrated that previous occupancy of an ICU room by a patient with multidrug-resistant (MDR) gram-negative bacteria is a risk factor for acquisition by subsequent occupants, others have infrequently recovered gram-negatives from the hospital environment [26,27]. Additionally, while contact precautions have been demonstrated to have efficacy in control of transmission of MRSA, VRE and *C. difficile*, the evidence is less robust for gram-negative organisms [28,29]. Although gram-negatives are the predominant pathogens in cases of ventilator-associated pneumonia and in ICU HAIs, where death from HAI is most likely to occur, existing guidelines pertaining to control of MDR pathogens either exclude gram-negatives or acknowledge limitations in recommendations pertaining to these organisms [30-32]. Increased ability to detect environmental reservoirs of these organisms should lead to improvements in targeted control efforts. Prior studies have evaluated (by TCM) the frequency of microorganisms on high-use surfaces, HCW, PPE, and other items in the healthcare environment, with widely differing results depending on the site sampled and organism of interest. Many have focused on one organism in the immediate surroundings of a patient known to be colonized with that organism [3,9,26,33-35]. Apart from this context, studies in non-outbreak settings often evaluate epidemiologically significant pathogens for one site of interest per study. One evaluation of MDR *A. baumannii* contamination in a medical intensive care unit found none except in colonized patients’ immediate surroundings [5]. In our institution, an evaluation (by TCM) was made of protective lead garments at various sites, with only 5 of 182 samples positive for any bacteria, all normal skin flora; another assessment of computer keyboards/mice recovered *S. aureus*, *Acinetobacter* spp., or *Pseudomonas* spp. on 17% of tested surfaces [2,4]. One study demonstrated a majority of bedside charts in the ICU were contaminated with MDR bacteria [36].

Against this backdrop, we sought to determine the burden and spectrum of microorganisms detected from HCW, PPE, and a variety of high-use hospital environmental surfaces by PCR/ESI-TOF-MS compared to TCM, and to compare the two methodologies in reference to the most commonly identified microorganisms detected among clinical cultures. This study is the first to our knowledge to evaluate selected sections of the hospital microbiome, comparing TCM and the unbiased T-5000-based PCR/ESI-TOF-MS method, in an effort to understand potential reservoirs of endemic nosocomial bacteria. Compared to traditional culture, PCR/ESI-TOF-MS detected more microbes, including more pathogens of potential clinical relevance, from a greater number of surfaces, hands of HCW, and PPE. PCR/ESI-TOF-MS also disproportionately recovered more gram-negative organisms missed by culture than *S. aureus*. As would be expected, both TCM and PCR/ESI-TOF-MS also detected many clinically less important organisms. Potentially clinically relevant pathogens accounted for similar proportions of all results (an estimated “signal-to-noise” ratio), unless streptococci, which are not typically considered major HAI pathogens, were included. However, compared to TCM, PCR/ESI-TOF-MS was able to detect potentially clinically relevant pathogens from 3-6x the number of sites screened, depending upon the inclusivity of the definition of “clinically relevant”. There is no standard definition for potential clinical relevance, and lower-virulence organisms, such as coagulase-negative staphylococci, can present major problems for patients with orthopedic or other implanted devices, or severely compromised patients. The study definition was chosen in order to reflect the most common pathogens seen clinically at the time and to include organisms for which there is high concern for virulence and/or drug resistance [32,37]. Additionally, much greater microorganism diversity was detected by PCR/ESI-TOF-MS, including unexpected organisms with high virulence or outbreak potential (e.g. *Clostridium tetani, S. pyogenes*). All surfaces, hands, and PPE samples demonstrated large numbers of recoverable pathogens, but without clear trends related to number of organisms by site. Based on these data, no obvious target for increased infection control efforts was seen in the study units. As this study was designed as a pilot study with a small number of sites/samples tested, there are clear limitations to the generalizability of the results to an entire hospital microbiome. It is also difficult to draw conclusions about organisms such as *Shigella flexneri* and *Borrelia turicatae* recovered from the hospital environment in the absence of known clinical cases during the study period. It is possible that these represent misidentifications of related organisms, as with hundreds of identified organisms, even 99% specificity would lead to several misidentifications. However, previous characterizations of this technology with 405 unique bacterial species have demonstrated accurate characterization in 95% of instances, with the remaining 5% unresolved species all accurate to the genus level [15]. Other limitations of the use of PCR/ESI-TOF-MS in this context include the possibility of detection of nonviable organisms, cost, the semi-quantitative nature of the data, and inability to recover specific strains linked to a patient or outbreak isolate. In this study, PCR/ESI-TOF-MS was not used to detect specific strains of bacteria detected, though it can be and has been used specifically for rapid genotyping of *A. baumannii*[21], *S. aureus*[22] and *Streptococcus pneumoniae*[38]. Thus, this technology may yet prove useful in outbreak investigations using environmental sampling, especially since it detected 4–5 fold higher numbers of pathogens per site without adversely affecting the ratio of clinically irrelevant microbes.

Interestingly, PCR/ESI-TOF-MS detected widespread resistance elements throughout the hospital environment. In the case of *mec*A, most were associated with CNS codetections rather than *S. aureus*. As *mec*A elements are widely distributed in CNS, this is not surprising [39]. *Van*A was not detected with enterococcus by PCR/ESI-TOF-MS, but did have an association with the presence of *Lactobacillus* spp. While this organism is largely intrinsically resistant to vancomycin, previous studies have demonstrated that this is not related to the presence of *van*A, which to our knowledge has not been demonstrated in lactobacilli [40,41]. It is interesting that one of the samples positive for *van*A, but without enterococci by PCR/ESI-TOF-MS, grew a susceptible *Enterococcus* spp. on clinical culture. Furthermore, none of the five samples positive for enterococci by TCM had this organism codetected by PCR/ESI-TOF-MS, generating a question of whether there might have been specific *Enterococcus* spp. detection problems, which have not been previously described. Overall, there were no attempts to resolve discordant results from TCM and PCR/ESI-TOF-MS, given that essentially every sample showed discordance, at least in greater number of pathogens isolated by the latter method. The PCR/ESI-TOF-MS technology does not screen for all possible resistance genes, and as applied here only detects the resistance element, without reporting whether it is incorporated into the genome of an organism. If more than one organism is present in the test sample along with the resistance element, it was not clear with which organism the element might be associated, if any. It is possible that free-floating or promiscuous plasmids are responsible for some of these detections, which also has significance for infection transmission in recent literature [42]. Given the difficulty of performing plasmid genetic analysis and whole genome sequencing of unculturable organisms, it is likely that horizontal transmission and intergenus transfer of antimicrobial resistance elements plays a larger role in healthcare-associated transmission of gram-negative MDR pathogens than has yet been described [43]. The ability of PCR/ESI-TOF-MS to screen for a broad spectrum of genetic elements may be a starting point for hypothesis development related to horizontal transmission.

## Conclusions

In summary, PCR/ESI-TOF-MS detected larger numbers and a greater diversity of organisms from a higher proportion of environmental surfaces in the hospital pilot study, particularly pathogenic gram-negative organisms, without adversely affecting the “signal-to-noise” ratio of common skin contaminants detected. This may prove to be a useful technology for investigations of hospital outbreaks. However, though PCR/ESI-TOF-MS has the capacity to genotype organisms, its use in this screening context did not provide for further information about strain or antimicrobial resistance. Additionally, further investigation is warranted in reference to the frequent detection of resistance elements, particularly *van*A, in the absence of known host species for these resistance elements. PCR/ESI-TOF-MS may be a useful adjunct among infection control investigational tools for understanding transmission of endemic pathogens.

## Competing interests

The authors declare that they have no competing interests.

## Authors’ contributions

*Study concept and design:* HY, JW, CM. *Acquisition of samples:* HY, MC. *Laboratory analyses:* RK, GE, CG, TS, JC. *Analysis and interpretation of data:* HY, RK, GE, CM. *Drafting of the manuscript:* HY, CM. *Critical revision of the manuscript for important intellectual content*: RK, GE, HC, KC, JW, JH, JC, KM. *Statistical analysis*: HY. All authors read and approved the final manuscript.

## Authors’ information

We give thanks for the life of collaborator, coauthor and friend J. William Costerton, who passed away during the final preparation of this manuscript.

## Disclaimer

The opinions or assertions contained herein are the private views of the authors and are not to be construed as official or reflecting the views of the Department of the Army, Department of the Air Force, Department of Defense or the U.S. Government. This work was prepared as part of their official duties and, as such, there is no copyright to be transferred. All authors report no conflicts of interest relevant to this article.

## Pre-publication history

The pre-publication history for this paper can be accessed here:

http://www.biomedcentral.com/1471-2334/12/252/prepub
